# Fluorescent ureteral catheters in laparoscopic surgery for rectal cancer with invasion of the uterus: A case report

**DOI:** 10.1016/j.amsu.2022.104114

**Published:** 2022-06-29

**Authors:** Takahiro Kitagawa, Shunjin Ryu, Atsuko Okamoto, Rui Marukuchi, Keigo Hara, Ryusuke Ito, Yukio Nakabayashi

**Affiliations:** Department of Digestive Surgery, Kawaguchi Municipal Medical Center, 180, Nishiaraijuku, Kawaguchi City, Saitama, 333-0833, Japan

**Keywords:** Fluorescent ureteral catheters, Rectal cancer, Uterus, Laparoscopic surgery, Case report

## Abstract

**Introduction:**

Resection of the uterus is required in some cases of colorectal cancer with invasion of the uterus. Localisation of the ureters to prevent ureteral injuries is important during resection of advanced colorectal cancer and combined resection of the uterus.

**Case presentation:**

We report a case of a woman in her 80s with rectal cancer with invasion of the uterus. She presented with appetite loss and lower abdominal pain. She was hospitalised after being diagnosed with intestinal obstruction due to rectal cancer. Colonoscopy revealed a tumor involving 100% of the circumference of the rectosigmoid colon, and imaging showed rectal cancer with invasion of the uterus and a giant uterine fibroid. Fluorescent ureteral catheters were placed bilaterally under cystoscopy, and laparoscopic anterior rectal resection, combined hysterectomy, and bilateral adnexectomy were performed 1 day later. Near-infrared visualisation of these catheters enabled safe release of the surrounding tissues from the uterus.

**Clinical discussion:**

Surgical treatment of rectal cancer with invasion of the uterus is not standardised and requires more complicated procedures, which are associated with a high risk of ureteral injury. Fluorescent ureteral catheters allow visualisation of the course of the ureters without releasing them, thereby enabling safe surgery.

**Conclusion:**

In fluorescence-guided surgery for rectal cancer, fluorescent ureteral catheters are particularly useful in patients with suspected invasion of other organs.

## Highlights

•T4b rectal cancer has higher risk of ureteral injury.
•Fluorescent ureteral catheter is visualised clearly via near-infrared light.
•Fluorescent ureteral catheter can be useful during surgery for T4b rectal cancer.

## 1 Introduction

Resection of the uterus is required in some cases of colorectal cancer with invasion of the uterus [1]. Laparoscopic surgery has been widely used in recent years, and laparoscopic multivisceral resection has been also performed for T4b colorectal cancer [2]. However, laparoscopic surgery for locally advanced cancer is difficult, so the indications should be determined while taking into consideration the skill of each surgical team [3]. Localisation of the ureters to prevent ureteral injuries is important during resection of advanced colorectal cancer. Here, we report a case of rectal cancer with invasion of the uterus that was safely treated with laparoscopic anterior rectal resection, combined hysterectomy, and bilateral adnexectomy by visualising the locations of the ureters with the Near-Infrared Ray Catheter (NIRC™) fluorescent ureteral catheter (NIRFUC; Nippon Covidien, Ltd., Tokyo, Japan). NIRFUC is a new catheter with built-in Near-Infrared Ray fluorescent resin. This case report has been reported in line with the SCARE Criteria [4].

## 2 Presentation of case

A woman in her 80s visited our hospital with chief complaints of appetite loss and lower abdominal pain for 2 weeks, and she was hospitalised upon diagnosis of intestinal obstruction due to rectal cancer. She has a history of glaucoma and hypertension. She does not have a history of surgical intervention, drug allergy, and significant family history or psychosocial history. The physical examination showed tenderness in the lower abdomen and a mass in the left lower abdomen detected by palpation. Thoracoabdominal computed tomography (CT) confirmed rectal wall thickening with contrast enhancement, and uterine margin was not clear, which suggested invasive disease. It also found a uterine fibroid (65 mm × 61 mm) and a left ovarian cyst (55 mm × 50 mm), and metastatic lesions were not found in other organs. Colonoscopy found a type 2 tumor involving 100% of the circumference of the rectosigmoid colon, and passage of the colonoscope was not possible. Tubular adenocarcinoma (tub1) was then diagnosed by biopsy. Although uterine invasion was observed, it was considered that R0 resection was possible by combined resection of the uterus. We decided to perform surgery without preoperative chemotherapy or radiotherapy, considering that she was in her late 80s. Nutritional management and bowel control were performed before surgery. She had good adherence to treatment. NIRFUC were placed bilaterally under cystoscopy 1 day before surgery.

### 2.1 Surgical findings

We performed laparoscopic anterior rectal resection, combined hysterectomy, and bilateral adnexectomy by visualising the locations of the ureters with NIRFUC. The surgery was performed at our hospital, which is a district general hospital, by a senior gastroenterologist supported by a senior gynecologist.

A tumor in the rectosigmoid colon had invaded an extensive area of the uterus. The sigmoid colon was mobilised using a medial approach. The inferior mesenteric artery was resected at the root, and lymphadenectomy was performed. The left ovarian cyst was resected, and then the right, left, and dorsal sides of the rectum were released.

**Fig. 1:**
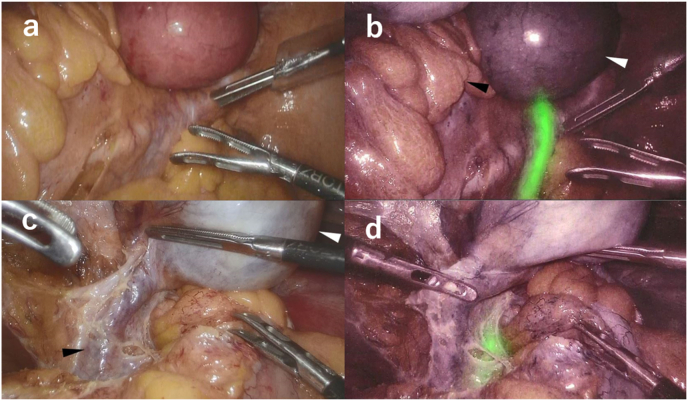
a: Conventional endoscopic view of the right side of the rectum before dissection. b: Fluorescence endoscopic view of the right side of the rectum before dissection. Fluorescence of the right ureter is clearly visible before dissection. The white and black arrowheads indicate the uterus and rectum, respectively. c: Conventional endoscopic view of left oophorectomy. The white arrowhead indicates the left ovarian cyst. The black arrowhead indicates the ovarian artery/vein. d: Fluorescence endoscopic view of left oophorectomy. Fluorescence of the left ureter is visible, which is helpful when ligating and dissecting the ovarian artery/vein.

**Fig. 2:**
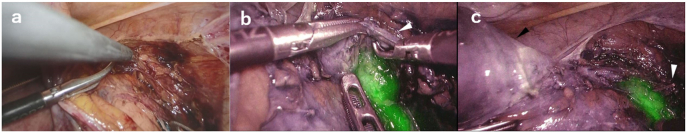
a: Conventional endoscopic view of the right uterine artery before separation, where the right ureter is not visible. b: Fluorescence endoscopic view of the right uterine artery before separation, where fluorescence of the right ureter is visible, allowing for safe dissection of the uterine artery. The white arrowhead indicates the right uterine artery. c: Fluorescence endoscopic view of the right uterine artery after dissection. The white arrowhead indicates the right uterine artery (after dissection). The black arrowhead indicates the uterus.

Due to unsuccessful transvaginal insertion of a uterine manipulator, the preparation of surgical field was difficult. However, the ureters were released safely from the uterus under near-infrared fluorescence visualisation of the ureteral catheters. The uterine arteries were clipped and resected. Because the surgical field in the pelvic area could not be secured due to the giant uterine fibroid, transection of the body of the uterus without cancer invasion and then the vagina was performed, followed by resection of the rectum with the part of the uterus with cancer invasion. Rectal anastomosis and vaginal suture closure were performed to complete the surgery. Intraoperative blood loss was 150 mL, and the duration of surgery was 9 h 13 min (Fig. 1, Fig. 2).

### 2.2 Postoperative course

A gastric ulcer and urinary disturbance occurred, which were resolved with oral medication. The patient was discharged 20 days after surgery. She did not want adjuvant chemotherapy, and surveillance for recurrence was scheduled at our outpatient clinic. Total colonoscopy could not be performed before the operation, so it was performed 3 months after the operation and ascending colon cancer was pointed out. Laparoscopic right hemicolectomy was performed 4 months after the operation. It has passed without recurrence until 6 months after the first operation.

### 2.3 Specimen images

**Fig. 3:**
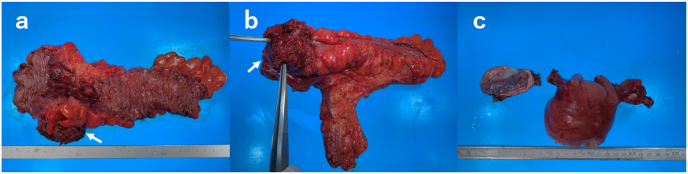
a: Gross appearance of the mucosal surface of the rectum. A 60-mm circumferential type 2 tumor is observed. b: Gross appearance of the serosal side of the rectum. Direct invasion of the uterine cervix by the tumor is seen (white arrow). c: The excised uterus and left ovary with a 70-mm fibroid and an ovarian cyst, respectively.

A type 2 tumor involving 100% of the circumference of the rectum was confirmed and was tightly adherent to the uterine cervix (Fig. 3).

### 2.4 Pathological findings

A type 2 tumor of 60 × 60 × 15 mm was found in the rectosigmoid colon. The tumor had invaded to the uterine cervix. The pathological results showed tub1, pN1a (1/21), BD2, Ly1a, V1b, Pn1a, INFa, pPM0 (130 mm), pDM0 (60 mm). The uterine margin was negative. A 70-mm leiomyoma was found in the uterus. A unilocular serous cyst was seen in the left ovary.

## 3 Discussion

Invasion of neighbouring organs has been reported in approximately 15% of patients with rectal cancer [5]. Gebhardt et al. [1] reported that multivisceral resection was required in 7% of patients with colorectal cancer, 14.5% (25/173) of whom underwent resection of the uterus. Colorectal cancer can be cured by multivisceral R0 resection even with invasion of neighbouring organs [1].

Ureteral injuries are significant complications of hysterectomy, as they occur in 0.73% of patients who undergo laparoscopic hysterectomy [6]. The surgical field is likely to be limited in cancer in which the uterus has been invaded; thus, special care is necessary to prevent ureteral injuries when resecting the uterus in such cases.

Indocyanine green (ICG) is one of the fluorescence dyes. ICG is excited with near-infrared light and emits near-infrared light of longer wavelengths. It can be visualised using a near-infrared laparoscopic system. ICG has been used in fluorescence-guided surgery [7,8].

ICG has been used for intraoperative visualisation of the ureters [9,10]. Although ICG is directly injected into the ureters, the fluorescence intensity varies depending on the thickness of the surrounding tissue; for example, reduced intensity has been reported in obese patients [9]. Intravenously injected methylene blue has also been used to visualise the ureters [[11], [12], [13], [14]]. However, methylene blue emits weaker light than ICG [12] and has limitations, such as its contraindication in patients with impaired kidney function and varying light intensity with changes in urine flow in the ureters [11]. In addition, because methylene blue damages DNA, its carcinogenicity is a concern [15].

Recently, solid resin materials with the near-infrared properties of ICG have been developed [16]. NIRFUC used in this study contain this resin. The ureters into which the catheters were inserted were visualised on a monitor by near-infrared irradiation using a near-infrared camera associated with the laparoscopic system. The quantum yield of the near-infrared fluorescent resin is reported to be ≥ 20 times than that of ICG [16], and thus, they provide favourable intraoperative visualisation of the ureters.

During laparoscopic simple total hysterectomy, the risk of ureteral injuries is particularly high when dissecting the ovarian cardinal ligament because the ureters cross the uterine arteries. In general, a space between the ureters and the uterus is created when the uterus is exposed with a uterine manipulator, which prevents ureteral injuries. However, in this study, a uterine manipulator could not be inserted due to the giant uterine fibroid. Nevertheless, even in such a high-risk case of ureteral injuries, NIRFUC enabled visualisation and preservation of the ureters.

Release from the surrounding tissue and exposure of the ureters is considered to be the most valid strategy to ensure their preservation. However, NIRFUC allow visualisation of the course of the ureters without releasing them, thereby enabling safe surgery and a shorter operating time. There are urological complications associated with fluorescent ureteral catheter placement, but it is expected to be the same as conventional ureteral catheter placement.

Unlike routine simple total hysterectomy, surgical treatment of rectal cancer with invasion of the uterus is not standardised and requires more complicated procedures, which are associated with a high risk of ureteral injury. NIRFUC can be useful in fluorescence-guided surgery for patients with invasion of other organs.

## 4 Conclusion

We encountered a case in which NIRFUC were useful in laparoscopic surgery for rectal cancer with invasion of the uterus. NIRFUC can be useful for preventing ureteral injuries in the treatment of rectal cancer with invasion of other organs.

## Ethical approval

This study was conducted with the approval of the Ethics Committee of the Kawaguchi Municipal Medical Center (approval number: 2020–3).

## Sources of funding

The authors received no funding sources for this article.

## Author contribution

Takahiro Kitagawa: Data Curation, Formal analysis, Project administration, Writing - Original Draft, Writing - Review & Editing. Shunjin Ryu: Data Curation, Writing - Review & Editing, Project administration. Atsuko Okamoto, Rui Marukuchi, Keigo Hara, and Ryusuke Ito were involved in investigation. Yukio Nakabayashi supervised the writing of the manuscript.

## Registration of Research Studies

N/a.

## Guarantor

Shunjin Ryu accepts full responsibility for the work, had access to the data, and controlled the decision to publish.

## Patient perspective

Although the patient was anxious at the time of admission, she understood the need for surgery and agreed to surgical treatment. After the operation, she gradually recovered and was happy.

## Consent

Written informed consent was obtained from the patient for publication of this case report and accompanying images. A copy of the written consent is available for review by the Editor-in-Chief of this journal on request.

## Provenance and peer review

Not commissioned, externally peer-reviewed.

## Declaration of competing interest

The authors declare no conflicts of interest.

## References

[1] Gebhardt C., Meyer W., Ruckriegel S., Meier U. (1999). Multivisceral resection of advanced colorectal carcinoma. Langenbeck's Arch. Surg..

[2] Zhang Xubing, Wu Qingbin, Gu Chaoyang, Hu Tao, Liang Bi, Wang Ziqiang (2019). Comparison of short and long-time outcomes between laparoscopic and conventional open multivisceral resection for primary T4b colorectal cancer. Asian J. Surg..

[3] Hashiguchi Y., Muro K., Saito Y., Ito Y., Ajioka Y., Hamaguchi T. (2020). Japanese Society for Cancer of the Colon and Rectum (JSCCR) guidelines 2019 for the treatment of colorectal cancer. Int. J. Clin. Oncol..

[4] Agha R.A., Franchi T., Sohrabi C., Mathew G., Kerwan A., Scare Group (2020 Dec). The SCARE 2020 guideline: updating consensus surgical CAse REport (SCARE) guidelines. Int. J. Surg..

[5] Sugarbaker P.H., Corlew S. (1982). Influence of surgical techniques on survival in patients with colorectal cancer. Dis. Colon Rectum.

[6] Adelman M.R., Bardsley T.R., Sharp H.T. (2014). Urinary tract injuries in laparoscopic hysterectomy: a systematic review. J. Minim. Invasive Gynecol..

[7] Schaafsma B.E., Mieog J.S., Hutteman M., van der Vorst J.R., Kuppen P.J., Löwik C.W. (2011). The clinical use of indocyanine green as a near-infrared fluorescent contrast agent for image-guided oncologic surgery. J. Surg. Oncol..

[8] Marshall M.V., Rasmussen J.C., Tan I.C., Aldrich M.B., Adams K.E., Wang X. (2010). Near-infrared fluorescence imaging in humans with indocyanine green: a review and update. Open Surg. Oncol. J..

[9] Siddighi S., Yune J.J., Hardesty J. (2014). Indocyanine green for intraoperative localization of ureter. Am. J. Obstet. Gynecol..

[10] Mandovra P., Kalikar V., Patankar R.V. (2019). Real-time visualization of ureters using indocyanine green during laparoscopic surgeries: can we make surgery safer?. Surg. Innovat..

[11] Verbeek F.P., van der Vorst J.R., Schaafsma B.E., Swijnenburg R.J., Gaarenstroom K.N., Elzevier H.W. (2013). Intraoperative near infrared fluorescence guided identification of the ureters using low dose methylene blue: a first in human experience. J. Urol..

[12] Barnes T.G., Hompes R., Birks J., Mortensen N.J., Jones O., Lindsey I. (2018). Methylene blue fluorescence of the ureter during colorectal surgery. Surg. Endosc..

[13] Al-Taher M., van den Bos J., Schols R.M., Bouvy N.D., Stassen L.P. (2016). Fluorescence ureteral visualization in human laparoscopic colorectal surgery using methylene blue. J. Laparoendosc. Adv. Surg. Tech..

[14] Matsui A., Tanaka E., Choi H.S., Kianzad V., Gioux S., Lomnes S.J., Frangioni J.V. (2010). Real-time, near-infrared, fluorescence-guided identification of the ureters using methylene blue. Surgery.

[15] Hiraku Y., Goto H., Kohno M., Kawanishi S., Murata M. (2014). Metal-mediated oxidative DNA damage induced by methylene blue. Biochim. Biophys. Acta.

[16] Anayama T., Sato T., Hirohashi K., Miyazaki R., Yamamoto M., Okada H. (2020). Near-infrared fluorescent solid material for visualizing indwelling devices implanted for medical use. Surg. Endosc..

